# Together We can Figure It out: Groups Find Hospitality Robots Easier to Use and Interact With Them More than Individuals

**DOI:** 10.3389/frobt.2021.730399

**Published:** 2021-10-25

**Authors:** Harrison Preusse, Rebecca Skulsky, Marlena R. Fraune, Betsy Bender Stringam

**Affiliations:** ^1^ Department of Psychology, Intergroup Human-Robot Interaction (iHRI) Lab, New Mexico State University, Las Cruces, NM, United States; ^2^ Department of Psychology, New Mexico State University, Las Cruces, NM, United States; ^3^ Hotel Restaurant & Tourism Management, New Mexico State University, Las Cruces, NM, United States

**Keywords:** human-robot interaction, group dynamics, group type, field studies, hospitality and tourism

## Abstract

As robots are becoming more prevalent and entering hospitality settings, understanding how different configurations of individuals and groups interact with them becomes increasingly important for catering to various people. This is especially important because group dynamics can affect people’s perceptions of situations and behavior in them. We present research examining how individuals and groups interact with and accept a humanoid robot greeter at a real-world café (Study 1) and in an online study (Study 2). In each study, we separately examine interactions of individuals, groups that participants formed after they arrived at the café (new-formed groups), and groups that participants arrived with at the café (pre-formed groups). Results support prior findings that groups are more likely to interact with a public robot than individuals (Study 1). We also report novel findings that new-formed groups interacted more with the robot than pre-formed groups (Study 1). We link this with groups perceiving the robot as more positive and easier to use (Study 2). Future research should examine perceptions of the robot immediately after interaction and in different hospitality contexts.

## Introduction

**FIGURE 1:**
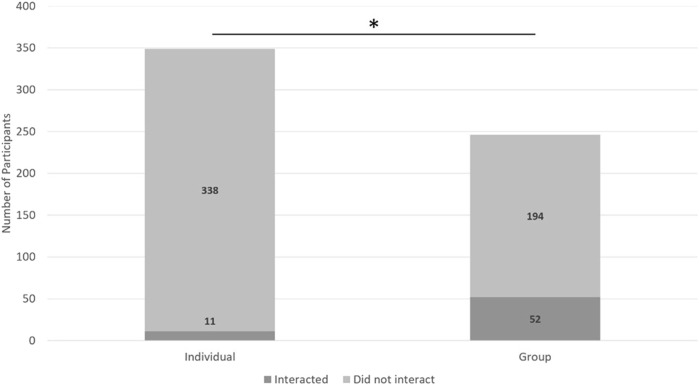
A description of the results from H1a. Asterisks indicate groups were more likely to verbally interact than individuals.

Technology is changing the process and product of guest service for the hospitality industry. Hotels and restaurants deliver a product that is part service and part tangible creation. Traditionally, a human has rendered service. However, advancements in robotics now allow robots and other technologies to render many hospitality services (e.g., Figure 1; Ivanov et al., 2017; Stringam and Gerdes, 2017). Robots can deliver towels to a hotel room, flip burgers, and raise and lower french fry baskets in a restaurant (Stringam and Gerdes, 2017). In some hotels, robots can check-in guests, answer concierge-type questions, and deliver luggage (Pierce, 2015). Despite these capabilities, robots are not common in hospitality businesses.

Consumer acceptance is one of many factors influencing robot adoption. Most hospitality services are produced and consumed simultaneously, with customers taking part in the process. The increased role of customers as part of the hospitality service delivery process places a higher requirement on consumer acceptance and willingness to use and interface with the technology (Kazandzhieva and Filipova, 2019).

Consumers’ willingness to use robots in service encounters often depends on prior positive interactions with robots (Chi et al., 2021). For the hospitality industry, this presents a cyclical obstacle: the low number of robots used by the hospitality industry results in a lack of familiarity with robots, which leads to less likelihood of prior consumer engagement with robots in the service setting, yielding a lack of propensity for consumer interaction or acceptance, resulting in a low adoption rate of robots, which then returns to a low number of robots in hospitality businesses.

While hospitality businesses are exploring the use of robots in service settings, studies examining the human-robot interactions for the hospitality industry are sparse (Chan and Tung, 2019; Collins, 2020; de Kervenoael et al., 2020; Tung and Law, 2017; Yu and Ngan, 2019). Most of the research on hospitality robots focuses on controlled laboratory experiments. To better understand and optimize the use of robots in hospitality settings, it is critical to study them in real world settings. Consumers often interact differently in real hospitality environments than in laboratory settings. Additionally, conducting studies in a live hospitality setting can help to discover new variables or unexpected patterns which may merit further study (Fraune et al., 2015; Šabanovic et al., 2011).

Hospitality products and services are often experienced in social settings, with groups of friends, family, or co-workers, bringing an additional, critical factor in human robot interaction. Real-world studies find that groups tend to interact with the robots far more than individuals (Kanda et al., 2004; Sabanovic et al., 2006). Often, when one person interacts with the robot, it draws the others of their group to interact with it (Fraune et al., 2019). Although this finding has been replicated many times, the underlying psychological reasons for this increased interaction are unclear.

Answers may come from social psychological theory, which typically applies to and offers good initial working hypotheses for HRI research (Bartneck et al., 2020; Groom and Nass, 2007; Reeves and Nass, 1997). Groups are often a protective factor for individuals, relieving stress, providing support (Haslam et al., 2019; Häusser et al., 2012), and even making people bolder, especially in competitive contexts (Insko et al., 1988; Wildschut et al., 2003). This is especially true (Brewer et al., 2004; Insko et al., 2013) for groups that are more unified, cohesive, or perceived by outsiders as “entitative” (Campbell, 1958), such as pre-existing groups (family, friends, and even coworkers), as opposed to new groups (collections of individuals who happen to be in the area; Lickel et al., 2001; Lickel et al., 2000). As a result, interacting with in-the-wild robots in groups (as opposed to individuals) may improve aspects of technology acceptance, such as perceived ease-of-use and self-efficacy. This comfort level for using the robot may in turn improve perceived usefulness, attitudes about using the robot, and intentions to use it. These effects may especially be true for pre-existing groups rather than new groups, because they are likely to be higher in group cohesion. So far, one study with a mall guidance robot in Japan has provided initial support for cohesive or pre-existing groups behaviorally interacting with the robot more, and giving more positive survey responses about the robot, compared to individuals (Fraune et al., 2019). However, the prior study did not examine how groups affect aspects of technology acceptance. Additionally, more research is needed on the effect of pre-existing groups on interactions with hospitality robots and with other robots in the wild and other countries.

The COVID-19 pandemic brought some extenuating circumstances to the present study. COVID-19, formally known as SARS-Cov-2, began December 2019, in Wuhan, China and spread rapidly around the world (González-Rodríguez and Labad, 2020). To reduce the spread of COVID-19 and reduce crowding at medical care facilities (Luchetti et al., 2020), national and international institutions ordered social distancing (standing at least 6 feet apart) and required people to wear masks (Giallonardo et al., 2020). Because of these guidelines, the restaurants were less busy than usual, and people wore protective face masks covering their nose and mouth. We expect that the level of activity at the restaurants is like off-peak times, making the result still relevant to businesses during times without pandemics. Because of the pandemic, people may have been less likely to interact with or stand near other people, especially those who were outside their groups. The pandemic lasted over a year, and science must go on, and so we continued the study despite these constraints.

## Materials and Methods

### Study 1: In-The-Wild Study

For Study 1, we examined how people interacted with a robot that was placed outside a restaurant.

#### Description of 100 West

We observed people interacting with a robot at the 100 West Café, an on-campus restaurant. The 100 West Café is a restaurant laboratory class for students in Hotel, Restaurant, and Tourism Management classes. Instructors manage the students who run the café as cooks, servers, and hosts. The restaurant is located in a classroom and office building on the campus and is open to the public from 12–1 pm for lunch, and patrons may come for dine-in or takeout.

#### Robot Platform: Pepper

**FIGURE 2:**
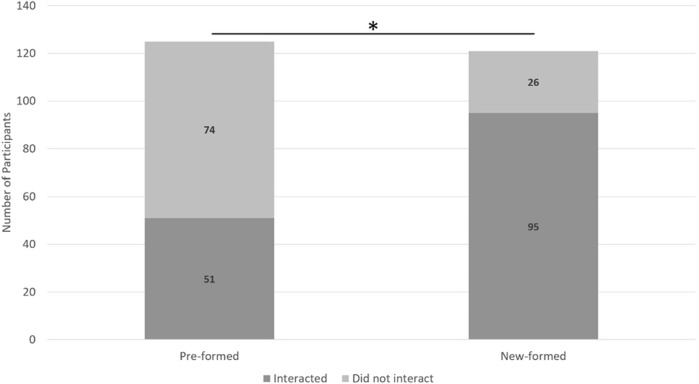
A description of the results from H2a. Asterisks indicate new-formed groups were more likely to verbally interact than pre-formed groups.

Pepper is a humanoid robot that was developed by Softbank Robotics, and stands at 4 ft (1.2 m) tall with a touch screen tablet on the chest. Pepper recognizes faces and basic human emotions, and Pepper comes with software that allows researchers to program various interactions (Figure 2).

Pepper stood outside the restaurant, and, for the first 4 days of the study, served as a host through which patrons could check in. On day 5, we changed Pepper’s role to a greeter/entertainer for guests waiting outside or entering the restaurant. We changed Pepper’s role because we realized the usage of face masks limited how participants could interact with Pepper–specifically, Pepper was unable to recognize faces when participants wore masks, and was therefore unable to respond to many of the participants’ verbal interactions. By changing Pepper’s role to a greeter/entertainer, the experimenters could initiate and control Pepper’s interactions, allowing conversations between participants and Pepper. This role change was evenly balanced across qualitative dependent variables.

#### Number of Days Observed

We gathered video feed data for 9 separate days while the restaurant operated. Cameras in the hallway recorded people’s interactions with the Pepper robot.

#### Participants

We noted 346 participants across all the videos. We did not attempt to code gender or age because we did not wish to assume and because all restaurant patrons wore face masks, which would make estimations even more difficult. Participants included university students, staff, and professors, and community members. All participants were restaurant patrons (*N* = 127), student workers (*N* = 34), or neither–henceforth referred to as bystanders (*N* = 185). Those involved with or who had knowledge about the details of the project (i.e., the researchers, head of the hospitality department, the chef) were not counted as participants. We defined people as new participants the first time they entered a video frame each day; if they reentered the video frame during the same day, we coded them under the same participant number. We did not track participants over multiple days because masks made it too difficult to recognize them.

#### Procedure

Both experimenters and a research assistant went to every data collection event. One experimenter controlled Pepper via a computer and initiated interactions as described below, while the second experimenter aided in notifying the first experimenter when new participants entered the hallway and gave input on which script to initiate. Because the setting was a restaurant, people’s arrival could not be controlled, and often several people or groups of people arrived at the same time. Thus, it was necessary to have two experimenters and an additional research assistant.

**FIGURE 3:**
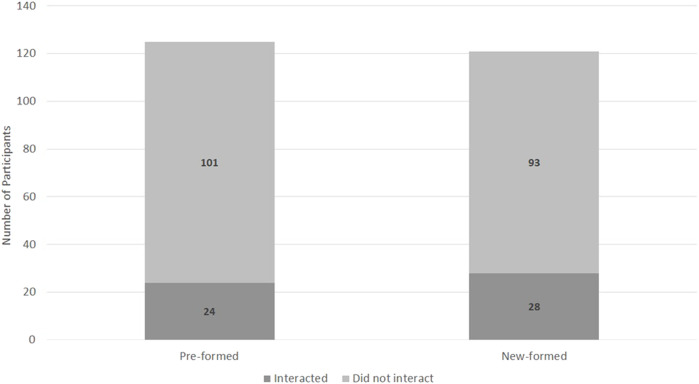
A description of the results from H2b.

Experimenters manually controlled Pepper’s verbal interactions and movement from a program called Choreographe Suites, from Softbank Robotics. In Pepper’s role as a host, Pepper’s script involved asking people if they were here for dine-in or takeout and asking them to check in on Pepper’s tablet, including information about the number of people in the party, and if they would dine-in or take-out. In Pepper’s final role as a greeter/entertainer, Pepper’s script involved two phases: phase one: Before the restaurant opened, Pepper asked people to socially distance and engaged them with general prompts. The experimenters chose these prompts at random and used as many as possible while participants were in the hallway. Phase two: Once the restaurant opened, Pepper asked participants if they were here for dine-in or take out, and then engaged them with the same general prompts. Because people only interacted with the robot either in phase one or phase two, we used the same prompts and random selection for each phase. We describe all Peppers interactions in Figure 3.

#### Measures

We identified variables of interest from the characteristics of participants and their interactions. Three research assistants manually coded participants as **individuals**, members of a **new-formed group**, or members of a **pre-formed group**. The coding of some participants changed through the duration of the video, such as when a participant entered as an individual but then became a member of a new-formed group. These research assistants additionally coded participants’ role as student worker, patron, or bystander. Finally, they coded the number of times and different ways participants interacted with Pepper: stop and observe, verbal interactions, non-verbal interactions, ignore Pepper–prompted, and ignore Pepper–not prompted (See Table 1 for all variable definitions).

**TABLE 1:** A description of all variable definitions used when coding the video feed and inter-rater reliability (IRR).

Variables	Categories	Description of code	IRR
**Group**	Individual	A participant who either: - Is not present with anyone with whom they have verbally interacted - Is not verbally interacting with anyone	0.90
	Group New-formed	A participant or a group member of the participant who either: - Is verbally interacting with someone with whom they did not arrive or dine - Is present with someone with whom they verbally interacted, with whom they did not arrive or dine	0.81
	Group Pre-formed	A participant who either: - Is verbally interacting with someone with whom they arrived - Is verbally interacting with someone with whom they would dine -Is present with someone with whom they would dine	0.90
**Role**	Student Worker	Chef or server working at 100 W (dressed in the uniform/dress of a chef/server).	0.92
	Patron	Someone who is dining or picking up food from 100 W. Someone who is not dining or	0.94
	Bystander	picking up food from 100 W	0.95
**Type of interact-ion**	Verbal Interaction	Saying a phrase or more directed at Pepper A body movement or gesture directed at	0.80
	Non-Verbal Interaction	Pepper (e.g., waving, moving hand in front of face, touching screen, mimic pepper	0.82
	Ignore Pepper - prompted	movements) No verbal or non-verbal interaction with Pepper, despite that Pepper	0.63
	Ignore Pepper–not prompted	used one or more prompts No verbal or non-verbal interaction with Pepper, and Pepper used no prompts	0.91

We also attempted to administer a questionnaire after participants’ interaction with the robot to learn more about their acceptance of this technology as an individual or group member. However, it soon became clear that people at the café did not have the time or interest in taking the questionnaire. Therefore, instead, we ran Study two to collect online survey data.

### Study 2: Online Survey

For Study 2, we implemented an online survey that asked more in-depth questions about people’s perceptions of robots and technology in general.

#### Participants

We recruited 78 students from the New Mexico State University participant pool for an online survey. Participants were compensated with course credit for research participation.

#### Conditions

We manipulated one independent variable with three conditions (Group: Individual, Pre-formed group, New-formed group) between-subjects.

#### Procedure

After providing informed consent, participants completed an online Qualtrics survey on their personal computers or devices. The survey consisted of two halves: a simulated video interaction with Pepper as a restaurant greeter (Interaction), followed by a series of questions regarding participants’ experience of their interaction with Pepper (Reaction).

At the beginning of the Interaction portion, the survey instructed participants to imagine going to dine at a restaurant and encountering a robot at the door. Depending on condition, this scenario asked participants to imagine that they approached this robot alone (Individual), with a group of their friends (Pre-formed group), or after mingling and chatting with strangers waiting outside the restaurant (New-formed group). Participants engaged in a series of simulated video interactions with Pepper. In the videos, Pepper spoke, moved, and asked questions. Then, participants chose a response from a pre-defined list. Through predefined survey logic, Pepper ‘responded’ to their choice in the next video.

In the initial video, Pepper bowed to participants, welcomed them to the restaurant, and then asked if they planned to dine-in or take-out food. For this interaction, participants only had the option to respond that they were dining in. In the second video, Pepper asked participants to wait outside the restaurant until it was their turn to be seated, then asked if they would like to hear about the inspirations behind that day’s menu. Participants chose to respond “Yes” to Pepper, or to “ignore” Pepper. If the participant chose to listen to Pepper, they watched a third video in which Pepper explained the origins of that day’s cuisine before inviting them into the restaurant. If they chose to ignore Pepper, they did not see a third video. Participants then progressed to the Reaction portion of the survey.

In the Reaction portion, participants responded to a series of scales regarding both their prior interaction with Pepper and their perspective on technology and robots in general. Finally, participants provided demographic information (age, gender). We debriefed participants afterward.

##### Measures

###### Technology Acceptance Model

To evaluate participants’ experience of interacting with Pepper, and their willingness to interact with Pepper in future encounters, we administered the Technology Acceptance Model (TAM) questionnaire (Park, 2009; Davis, 1989). Participants indicated their agreement with 17 items across subscales of perceived ease-of-use (three items, e.g., “I find Pepper easy to use”), perceived usefulness (three items, e.g., “Pepper would improve my restaurant experience”), attitude (three items, e.g., “Checking in through Pepper is a good idea”), behavioral intention (two items, e.g., “I intend to be a heavy user of Pepper”), self-efficacy (two items, e.g., “I feel confident checking in with Pepper”), subjective norm (three items, e.g., “What Pepper stands for is important to me as a restaurant goer”), and system accessibility (one item: “I have no difficulty accessing and using Pepper for check ins”) on a scale from 1 (“Strongly disagree”) to 7 (“Strongly agree”). The survey prompted participants to respond based on how they perceived interacting with Pepper in their specific Group condition (Individual, Pre-formed group, or New-formed group). All other measures were the same regardless of condition.

We also measured participant anxiety about the robots (Nomura et al., 2006), threat from the robots (Fraune et al., 2015), and affinity for technology (Franke et al., 2019). However, because they do not relate directly to hypotheses in this paper, we do not report the results here.

### Hypotheses for Study one and two

We hypothesize:

#### H1: Members of Groups Will Interact More With Service Robots Than Individuals

H1a: Group members will have more *verbal* interactions with Pepper than individuals. (Study 1).
H1b: Group members will have more *non-verbal* interactions with Pepper than individuals. (Study 1).
H1c: Respondents assigned to group conditions will choose to interact with Pepper in a *simulated scenario* more than those assigned to individual conditions. (Study 2).

#### H2: Members of Different Types of Groups Will Interact With Service Robots in Different Ways

H2a: Members of pre-formed groups will have more *verbal* interactions with Pepper than members of new-formed groups. (Study 1).
H2b: Members of pre-formed groups will have more *non-verbal* interactions with Pepper than members of new-formed groups. (Study 1).
H2c: Respondents assigned to pre-formed group conditions will choose to interact with Pepper in a *simulated scenario* more than those assigned to new-formed group conditions. (Study 2).

#### H3: Members of Groups Are More Accepting of a Service Robot Than Individuals

H3a: Participants assigned to group conditions will indicate greater acceptance of Pepper in a simulated scenario on the TAM measure than those assigned to individual conditions. (Study 2).
H3b: Participants assigned to pre-formed group conditions will indicate greater acceptance of Pepper in a simulated scenario on the TAM measure than those assigned to new-formed group conditions. (Study 2).

## Results

Data were analyzed in JASP version 0.14.1. *p*-values of <0.05 were considered statistically significant.

### Study 1: In-The-Wild

#### H1: Members of Groups Are More Willing to Interact With Service Robots Than Individuals

H1a: Group Members Will Have More *Verbal* Interactions With Pepper Than Individuals. (Study 1)

**FIGURE 4:**
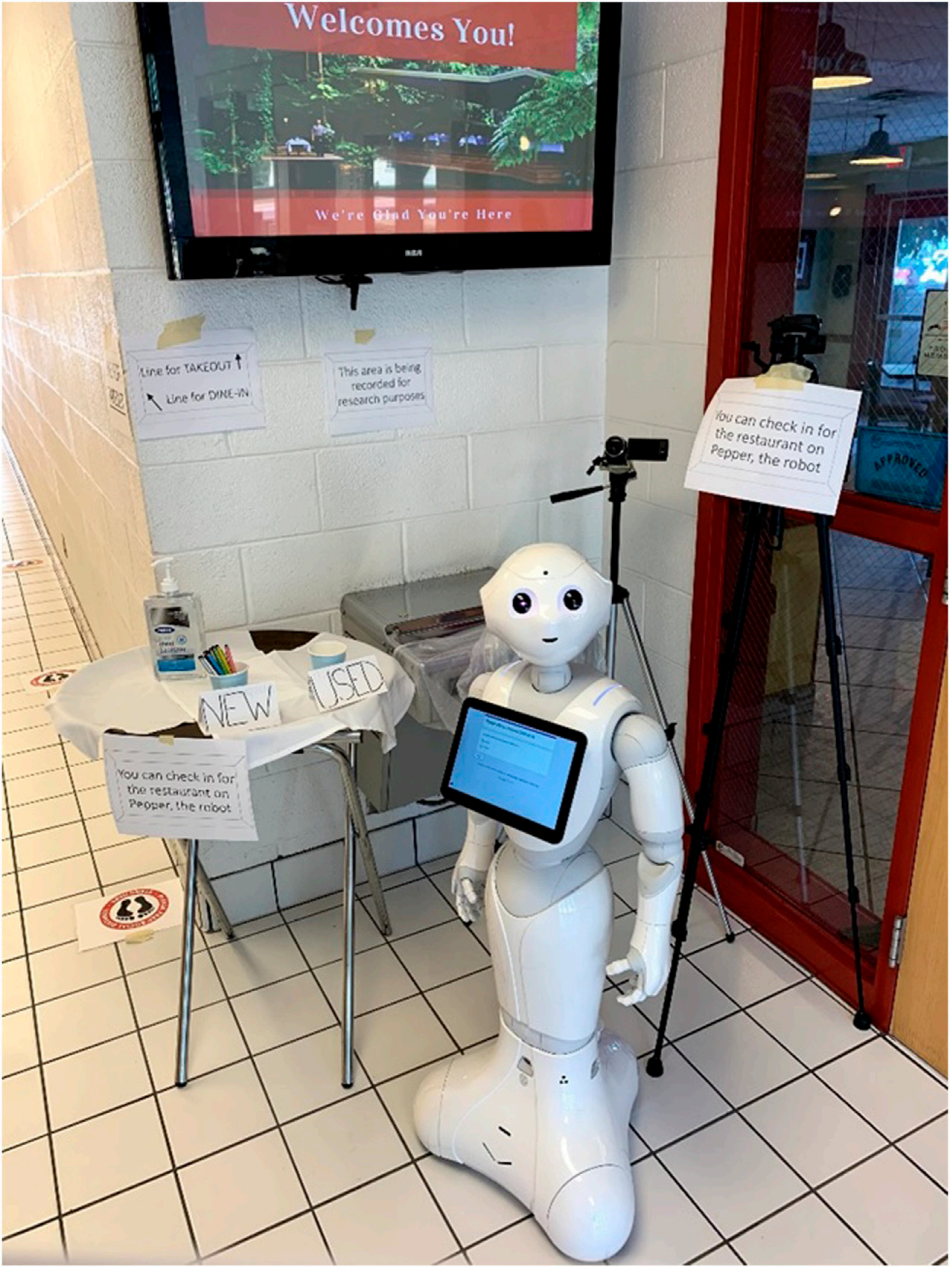
Pepper acting as a greeter at the 100 W. Café.

A 2 (individual or group) x 2 (verbally interact or did not verbally interact) chi-square test indicated a significant relationship between if someone belonged to a group or was an individual and if they verbally interacted with the robot (χ2 (2, *N* = 595) = 209.21, *p* < 0.01, *Crame*r’s V = 0.59) such that groups were more likely to verbally interact with Pepper than individuals (Figure 4).

##### H1b: Group Members Will Have More *Non-verbal* Interactions With Pepper Than Individuals. (Study 1)

**FIGURE 5:**
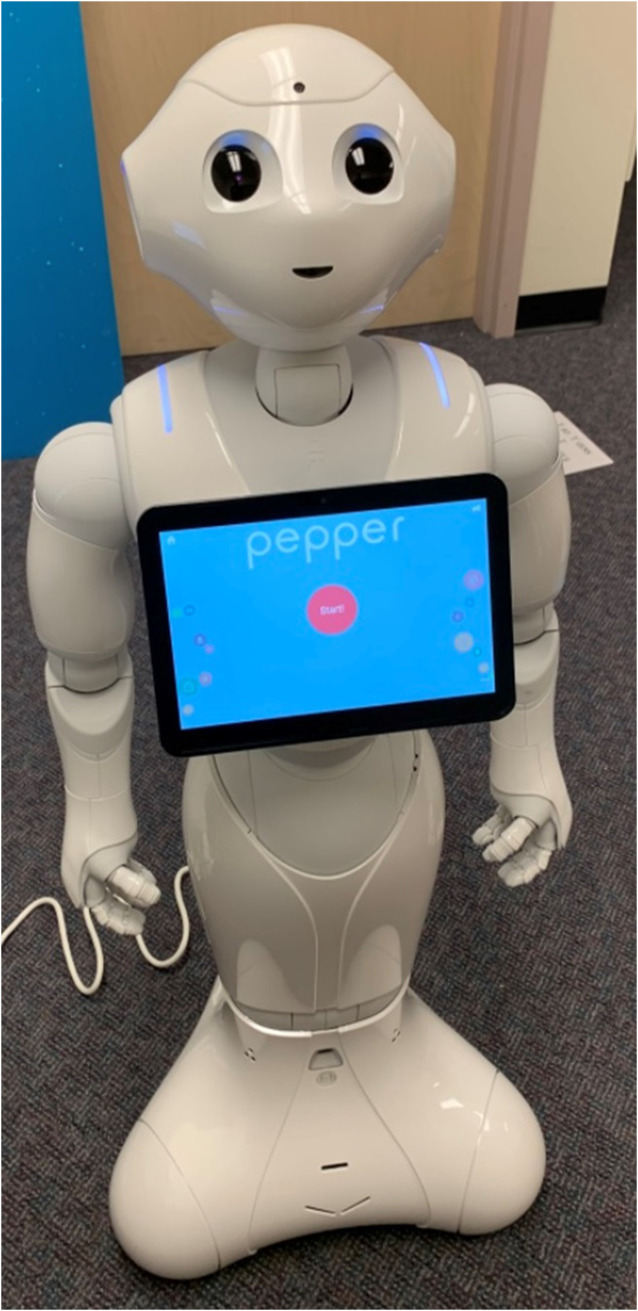
A picture of Pepper the robot from Softbank Robotics.

A 2 (individual or group) x 2 (non-verbally interact or did not non-verbally interact) chi-square test indicated a significant relationship between whether someone belonged to a group or was an individual and if they non-verbally interacted with the robot (χ^2^ (2, *N* = 595) = 49.31, *p* < 0.01, *Cramer’s V* = 0.29) such that groups were more likely to non-verbally interact than individuals (Figure 5).

##### H2: Members of Different Types of Groups Will Interact With Service Robots in Different Ways

H2a: Members of pre-formed groups will have more verbal interactions with Pepper than members of new-formed groups. (Study 1).

**FIGURE 6:**
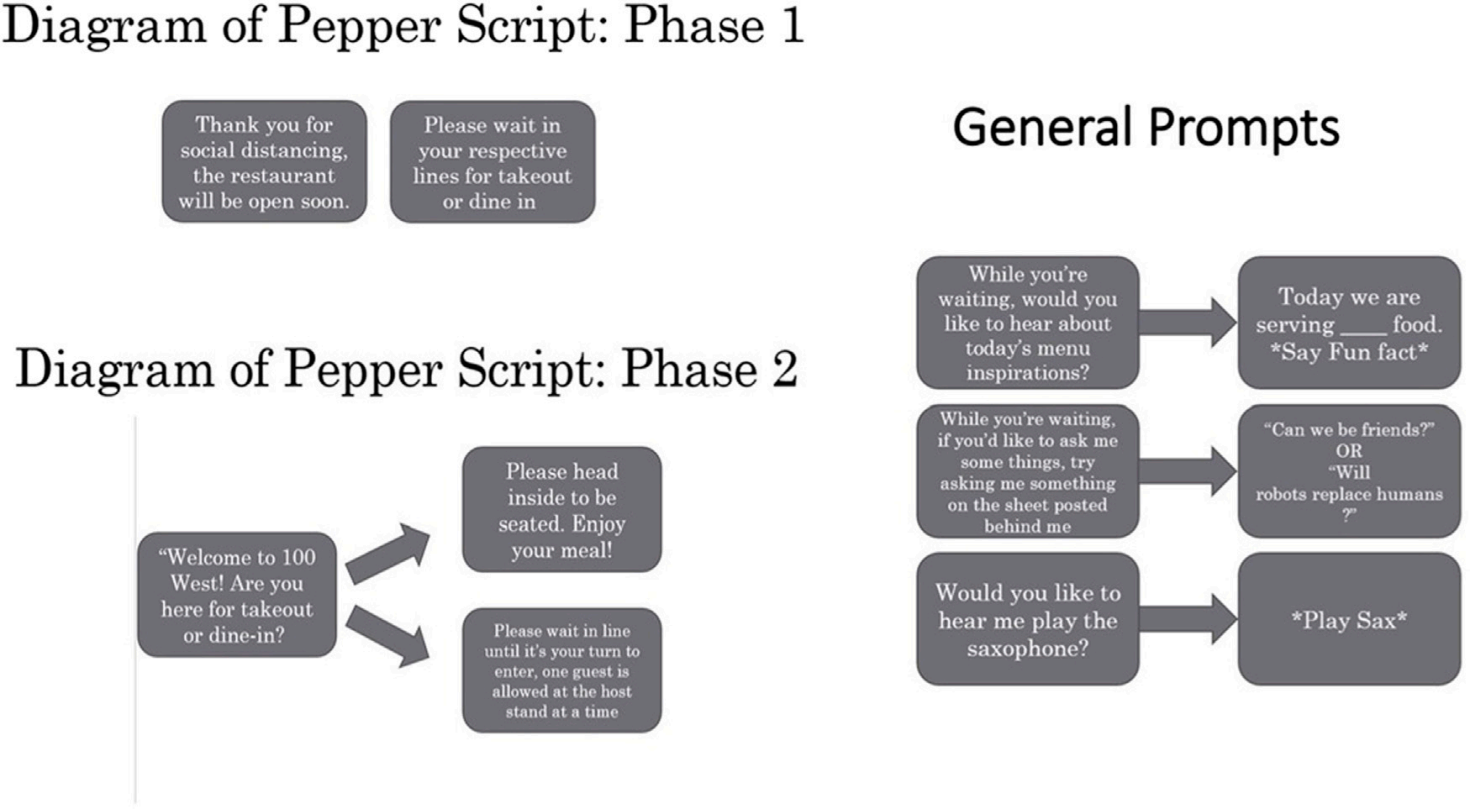
A depiction of all possible interactions with Pepper. Phase one and phase two scripts occurred in the first half and last half of days of data collection, respectively. General prompts occurred across phase one and phase two.

A 2 (Group Type: pre-formed or new-formed) x 2 (verbally interact or did not verbally interact) chi-square test indicated a significant relationship between group type and if they verbally interacted (χ^2^ (2, *N* = 246) = 36.25, *p* < 0.01, *Cramer’s V* = 0.38) such that new-formed groups were more likely to verbally interact than pre-formed groups (Figure 6).

H2b: Members of pre-formed groups will have more non-verbal interactions with Pepper than members of new-formed groups. (Study 1).

**FIGURE 7:**
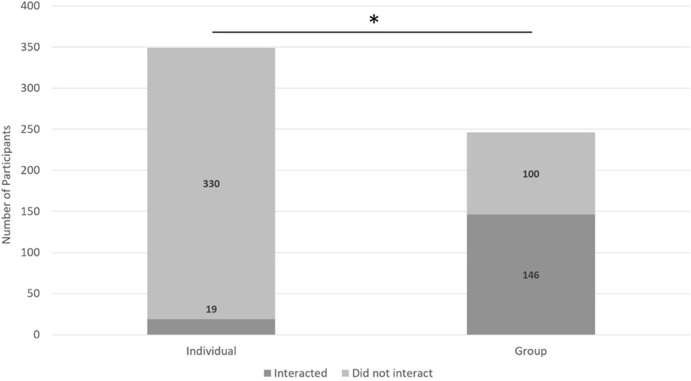
A description of the results from H1b. Asterisks indicate groups were more likely to non-verbally interact than individuals.

A 2 (Group Type: pre-formed or new-formed) x 2 (non-verbally interact or did not non-verbally interact) chi-square test indicated no significant relationship between group type and whether they non-verbally interacted or not (χ^2^ (2, *N* = 246) = 0.57, *p* = 0.45; Figure 7).

### Study 2: Online Survey

#### H1: Members of Groups Are More Willing to Interact With Service Robots Than Individuals

H1c: Respondents assigned to group conditions will choose to interact with Pepper in a simulated scenario more than those assigned to individual conditions. (Study 2).

A 2 (group or individual) x 2 (verbally interact or did not verbally interact) chi-square test indicated no significant relationship between if someone belonged to a group or was an individual and if they verbally interacted in a simulated scenario (χ^2^ (2, *N* = 114) = 0.04, *p* = 0.83; Table 2).

**TABLE 2:** Descriptive statistics for H1C and H2C.

Hypothesis	Condition	Interacted	Did not interact	Total
H1C: Online (Verbal)	Individual	40	6	46
	Group	60	8	68
H2C: Online (Verbal)	Pre-formed	22	5	27
	New-formed	38	3	41

#### H2: Members of Different Types of Groups Will Interact With Service Robots in Different Ways

H2c: Respondents assigned to pre-formed group conditions will choose to interact with Pepper in a simulated scenario more than those assigned to new-formed group conditions. (Study 2).

A 2 (group or individual) x 2 (non-verbally interact or did not non-verbally interact) chi-square test indicated no significant relationship between if someone belonged to a group or was an individual and if they verbally interacted in a simulated scenario (χ^2^ (2, *N* = 68) = 1.97, *p* < 0.17; Table 2).

#### H3: Members of Groups Are More Accepting of a Service Robot Than Individuals

H3a: Participants assigned to group conditions will indicate greater acceptance of Pepper in a simulated scenario on the TAM measure than those assigned to individual conditions. (Study 2).
H3b: Participants assigned to pre-formed group conditions will indicate greater acceptance of Pepper in a simulated scenario on the TAM measure than those assigned to new-formed group conditions. (Study 2).

**FIGURE 8:**
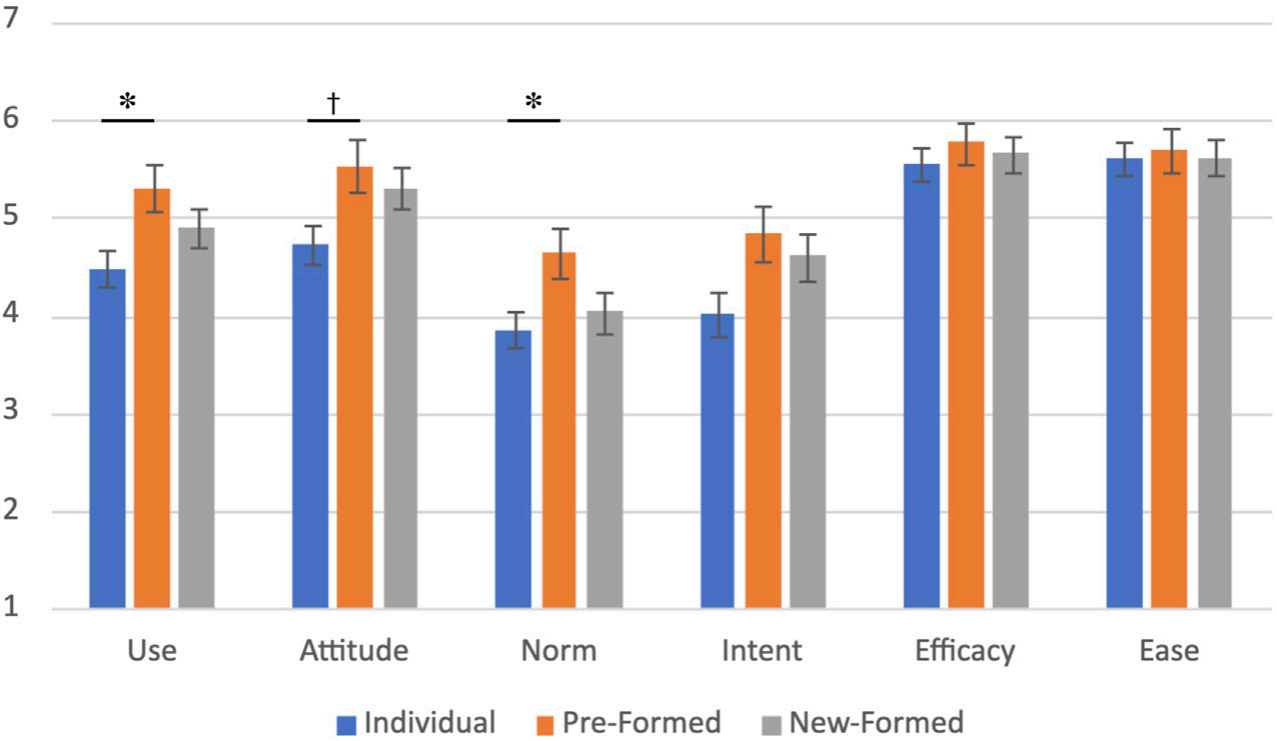
A description of the results from H3. Asterisks indicate that participants who imagined pre-formed groups had more positive perceptions of the robot than participants who imagined being individuals. Crosses indicate when the ANOVA had significance, and the posthoc test *p*-value was <0.1.

To test these hypotheses and account for variance, we ran one ANOVA (Individual x Pre-Formed x New-Formed) and post-hoc tests using Sidak corrections. On measures of use, attitude, and norm, the ANOVA indicated a significant difference across conditions. Specifically, participants in the pre-formed group condition rated the robot as more positive on usefulness (*M* = 5.30, *SD* = 1.42) and attitude (*M* = 5.53, *SD* = 1.28; marginally significant) and rated stronger norms for interacting with it (*M* = 4.64, *SD* = 1.54) then participants in the individual condition (usefulness *M* = 4.48, *SD* = 1.30; attitude *M* = 4.72, *SD* = 1.47; interacting with that *M* = 3.86, *SD* = 1.23). There was no significant difference between participants assigned to group or individual conditions on ease-of-use, self-efficacy, or intent scales (Table 3; Figure 8). Due to error, the ‘system accessibility’ subscale (only one item) was not collected for individual participants.

**TABLE 3:** Hypothesis 3–Technology Acceptance Measure (TAM) score by condition. We reported post hoc significance tests when the ANOVA was statistically significant and the post hoc *p*-value was less than 0.1.

	*F*	*p*	*η*_*p*_^2^	Individual x new-formed *p*	Individual x pre-formed *p*	New- x pre-formed *p*
Usefulness	3.61	0.030	0.061		0.027	
Attitude	3.44	0.036	0.058		0.052	
Norm	3.19	0.045	0.054		0.043	
Intent	3.03	0.052				
Efficacy	0.34	0.710				
Ease	0.05	0.955				

## Discussion

In this paper, we examined the effect of being in a group of different types on interaction with a humanoid robot in a restaurant service role in the wild (Study 1) and through simulated interaction (Study 2).

In Study 1, participants interacted with the Pepper robot while walking in the hallway, waiting for their takeout meal, or waiting to be seated in the restaurant. Groups were more likely than individuals to verbally and non-verbally interact with the robot (supporting H1a-b), which is consistent with previous literature (Fraune et al., 2019; Kanda et al., 2004; Sabanovic et al., 2006). Interestingly, we also found that new-formed groups were more likely than pre-formed groups to verbally interact with the robot (opposite of H2a).

In Study 2, participants completed an online questionnaire, including a simulated video interaction with Pepper while imagining that they were in one of three group conditions: interacting as an individual, as a member of a pre-formed group, or as a member of a new-formed group. Participants did not differ in their number of simulated verbal interactions with Pepper based on the type of group they imagined themselves to be in (failing to support H1c and H2c). While simulated interactions did not differ, participants imagining themselves in a pre-formed group self-reported more acceptance of Pepper than participants imagining themselves as individuals along three of the six dimensions of the Technology Acceptance Scale: perceived usefulness, attitude, and norms (partially supporting H3). We discuss this in more depth below.

### H1: Members of Groups Are More Willing to Interact With Service Robots Than Individuals

Our results support previous findings (Fraune et al., 2019; Kanda et al., 2004; Sabanovic et al., 2006) that groups interact more with robots in the wild than individuals. We found this during actual interaction (Study one; H1a-b), but not during the online questionnaire (Study two; H1c). This discrepancy between actual and online interaction is consistent with prior findings that users demonstrate a greater range of interactions when physically sharing space with an embodied robot rather than responding to videos of them (Bainbridge et al., 2011). We emphasize the importance of conducting real-world studies to examine complex phenomena like group dynamics, which rely on the group (not just the participant) for the full dynamics.

### H2: Members of Different Types of Groups Will Interact With Service Robots in Different Ways

Participants in new-formed groups interacted more with the robot than those from pre-formed groups. This contradicts prior findings of more cohesive groups interacting more with robots in the wild (Fraune et al., 2019). To reflect on similarities and differences between the present and this prior (Fraune et al., 2019) in-the-wild studies that focused on characteristics of groups, we discuss four possible factors contributing to the development of groups, group dynamics, and interaction with robots in the wild. In future studies, researchers should more specifically examine these factors to enhance our understanding of why and under what circumstances groups interact more with robots and individuals.

First, context matters. In locations with a higher concentration of people, it is more likely that people will form new groups. This can be likened to “loose associations” that prior psychological studies report about (e.g., groups made of people standing in line; Lickel et al., 2001; Lickel et al., 2000). This would explain why new groups did not typically form in the prior study (Fraune et al., 2019), but often formed in the present study, which had a higher concentration of people.

Second, the dimensions related to the robot itself contribute to interaction. Like in other studies, the robot can be a talking point or promote curiosity about how it functions (Fallatah et al., 2020; Law et al., 2017). In both studies, interest in these ways contributed to interactions with the robot.

Third, the presence of the robot can prompt humans to interact with other humans–that is, to form groups of humans. We reflect on two methods of this: (A) Work on group dynamics indicates that the mere presence of an outgroup member influences people to categorize themselves and others as ingroup members (Turner et al., 1987). In this context, the presence of a robot may cause people to perceive shared group membership with other humans, and therefore interact with them more (B) Interest in the robot as a talking point or about how it functions can prompt people to discuss this with other humans, as illustrated in other studies (Wada et al., 2010; Chang and Sabanovic, 2015).

Fourth, human groups promote interaction with the robots. We suggest two mechanisms for this: (A) When people interact with the robot, they create a social norm for interacting with it, which increases the likelihood that others will do so as well (Burger and Shelton, 2011; Cialdini, 2007; Smith et al., 2007)–especially in uncertain or novel contexts. The prior study found that if one human group member interacted with a robot, others from the same group were more likely to interact with it as well (Fraune et al., 2019) (B) Feeling group membership (pre-formed or new-formed) increases perceived safety–or at least decreases fear (Gaertner and Insko, 2000; Insko et al., 1990) and thereby changes perceptions of interaction. These changed perceptions can make people feel more comfortable interacting with the robot, thereby increasing the likelihood that they will interact with it. In the next section we discuss findings from Study two in which participants imagining themselves in a group rated more positive perceptions of the robot and of interacting with it.

### H3: Members of Groups Are More Accepting of a Service Robot Than Individuals

Our survey results support our discussion above about group membership changing perceptions of the robot. These results are most similar to results in the former study (Fraune et al., 2019), with participants who imagined being in pre-formed groups compared to those who imagined being individuals self-reporting more positive perceptions of Pepper along three of the six dimensions of the Technology Acceptance Scale (perceived usefulness, attitude, and norms). Participants who imagined themselves in new-formed groups had ratings in the middle of these, but differences were not statistically significant. Hospitality businesses can influence customer willingness to use robots by placing robots in social environments where groups are likely to exist or have the potential to form such as the lobby, a lounge, or a bar.

The reason these results were closely aligned with the former, rather than the present, study may be because participants imagine a situation more similar to that one than the present one with a high concentration of people. Survey results collected after the in-the-wild interaction may have paralleled the findings in the present study more closely. We discuss this limitation and recommendations for future research below.

### Limitations and Future Directions

One limitation of Study one was that the presence of the experimenters may have complicated whether there were any true individuals interacting with Pepper. Pepper was unable to utilize facial recognition because participants were wearing masks; thus, the experimenters needed to be in view and hearing of interactions to determine what script to play next to keep a natural flow of conversation between Pepper and the participants. The experimenters also needed to be close enough to intervene in case anyone mistreated the robots as in previous studies (Parasuraman and Riley, 1997; Brščić et al., 2015; Fraune et al., 2015). Therefore, participants were never truly alone, as the experimenters were always observing and sitting in the same area. Though the experimenters tried to remain as inconspicuous as possible, they may have influenced how people interacted with Pepper, prompting changes in interactions because strangers were present.

Originally, we sought to collect survey data from participants after their in-the-wild interaction with the Pepper robot. However, concerns over the transmission of the Covid-19 virus made participants less receptive to completing a paper study and limited participants’ willingness to complete an online survey, thus only two in-the-wild participants acquiesced to take the survey. Therefore, we collected survey responses online in a separate study. However, results indicated that the online survey did not accurately capture differences in interactions with individuals or groups. Therefore, this also calls into question the similarity between online survey responses and what in-person survey responses would have been. Researchers could try to create a more immersive online study environment by using platforms such as gather. town, in which multiple people can join the study at the same time to create a realistic sense of a group. Beyond the pandemic, it may be easier to obtain in-person survey responses, and we recommend that future research do so.

Limitations to restaurants and concerns of virus transmission during the Covid-19 pandemic may have impacted customers’ willingness and ability to interact with the robot and willingness to form new groups. We recommend that researchers replicate the study in two to 3 years, to measure and compare and responses post pandemic.

Another limitation to the study comes from the restaurant environment. Pre-formed groups and newly formed groups are not as distinct in a restaurant foyer or hallway entrance, or most hospitality settings. It is possible that customers who are dining together (pre-formed groups) arrived separately thus simulating a new-formed group. Further clouding group definition, it is also possible that a table or group of frequent patrons to the restaurant may already be acquainted with other groups of frequent patrons, making individuals of new-formed groups familiar to each other. These limitations warrant further study.

We changed Pepper’s nominal role at the restaurant from host to greeter early in Study one to accommodate mask-wearing users. However, we maintained Pepper’s physical location at the front of the restaurant, Pepper’s modes of interaction (verbal, touch), and the complexity of the conversation tree. The changes were balanced across individuals and groups of participants. Therefore, we do not believe that this change in role meaningfully affected the types of interactions that individuals or groups had with Pepper.

Researchers should run future studies in other hospitality environments. People dine and travel for many different reasons, in vastly different hospitality environments. Further, hospitality businesses are social settings, and group formation and composition differ among settings. These differences may impact results.

## Conclusion

In these studies, participants had a chance to interact with a robot outside a café (Study 1) or imagined themselves having that interaction (Study 2).

Study one strengthens previous findings in that groups are more likely to interact with a robot compared to individuals. It also presents a novel finding that the type of group participants are in influenced how likely they were to interact, and the kind of interaction they were likely to have, with the robot. Notably, members of new-formed groups had more frequent interactions with the robot than members of pre-formed groups. Designers should consider this when introducing robots into hospitality spaces. Hospitality environments, such as restaurants, bars, and hotels, may differ in the types of groups they cater to. For example, a social robot may encourage more interactions in a bar, a space conducive to forming new groups than a hotel, which may primarily cater to pre-formed groups. It is recommended that future studies explore how customer interactions with a robot vary with the location or setting in hospitality environments.

Study two also strengthens the idea that groups are more likely to interact with a robot, as those who imagined themselves in a group were more accepting of the robot than individuals. It provided novel insights that in a hospitality setting groups may perceive robots as more positive and useful than individuals perceive them. The pandemic accelerated the use of robots in hospitality service settings. Previously consumers were reluctant to consider robots as service, preferring the human touch (Murphy et al., 2017; Lin et al., 2020). This study helps the hospitality industry increase customer willingness to interact with service robots by employing robots in locations and functions that encourage group interaction, or where groups are more likely to exist. To encourage individuals to interact with robots, designers might investigate methods of detecting whether a user approaches alone or as part of a group, and then adapt its behavior accordingly. Hospitality robots could prioritize initiating interactions with individuals over groups, or encourage individuals to gather and form groups, thereby increasing the likelihood of an interaction. It is important to note that results from Study one and Study two cannot be directly compared because they used two different populations and settings.

Future research should also examine how underlying perceptions and motivations related to interacting with the robot change for different types of groups and influence their likelihood to interact with robots.

## Data Availability

The raw data supporting the conclusions of this article will be made available by the authors, without undue reservation.
